# Regional specificity in the subjective evaluation of factors determining health in Russians

**DOI:** 10.1192/j.eurpsy.2022.1408

**Published:** 2022-09-01

**Authors:** R. Shilko, L. Shaigerova, O. Almazova, A. Dolgikh, O. Vakhantseva

**Affiliations:** Lomonosov Moscow State University, Psychology, Moscow, Russian Federation

**Keywords:** factors determining human health, subjective evaluation, regional specificity, sociocultural determination

## Abstract

**Introduction:**

Important role of sociocultural mediation of human health is reflected in the regional specificity of subjective evaluation of factors determining human health.

**Objectives:**

The research is focused on the opinion of respondents from different regions of Russia about the importance of various factors that determine health and subjective well-being.

**Methods:**

210 men and 403 women aged 14 to 76 years (M=26.9; SD=13.7) from six regions of the Russia (Moscow, St. Petersburg, Udmurtia, Sakha, Sverdlovsk and Kemerovo) participated in the study. Respondents were asked to rank six factors (genetics, healthy lifestyle, good ecology, regular medical examination, absence of stress, financial well-being) in terms of their impact on health (1 is the most important, 6 is the least important).

**Results:**

It was revealed significant differences among respondents from different regions of the Russia in evaluation of importance of health factors such as “genetics” (F=3.317; p=0.003) and “good ecology” (F=5.008; p<0.001). Respondents from the Sverdlovsk consider “genetics” significantly more important than participants from the Sakha (MD=-0.706; p=0.019) and Kemerovo (MD=-0.859; p=0.015). Respondents from St. Petersburg consider the ‘good ecology’ significantly less important than participants from Moscow (MD=0.791; p=0.046), Udmurtia (MD=0.867; p=0.035), Sakha (MD=1.168; p<0.001), and Kemerovo (MD=1.286; p<0.001).

**Conclusions:**

Regional specificity was found in the subjective evaluation of the importance of factors that determine health and subjective well-being. The reported study was funded by the RFBR, project number 17-29-02506.

**Disclosure:**

No significant relationships.

